# *Cocculus hirsutus*-Derived Phytopharmaceutical Drug Has Potent Anti-dengue Activity

**DOI:** 10.3389/fmicb.2021.746110

**Published:** 2021-11-29

**Authors:** Rahul Shukla, Ravi Kant Rajpoot, Ankur Poddar, Richa Ahuja, Hemalatha Beesetti, Rajgokul K. Shanmugam, Shivam Chaturvedi, Kaushal Nayyar, Deepika Singh, Venugopal Singamaneni, Prasoon Gupta, Ajai Prakash Gupta, Sumeet Gairola, Pankaj Kumar, Y. S. Bedi, Tapesh Jain, Bhupendra Vashishta, Ravindra Patil, Harish Madan, Sumit Madan, Rinku Kalra, Ruchi Sood, Ram A. Vishwakarma, D. Srinivasa Reddy, Altaf A. Lal, Upasana Arora, Navin Khanna

**Affiliations:** ^1^Translational Health Group, Molecular Medicine Division, International Centre for Genetic Engineering and Biotechnology, New Delhi, India; ^2^Sun Pharmaceutical Industries Limited, Gurugram, India; ^3^Council of Scientific and Industrial Research (CSIR)-Indian Institute of Integrative Medicine, Jammu, India

**Keywords:** dengue, phytopharmaceutical, *Cissampelos pareira*, *Cocculus hirsutus*, AG129 mice, vascular leakage, TNF-α, IL-6

## Abstract

Dengue is a serious public health concern worldwide, with ∼3 billion people at risk of contracting dengue virus (DENV) infections, with some suffering severe consequences of disease and leading to death. Currently, there is no broad use vaccine or drug available for the prevention or treatment of dengue, which leaves only anti-mosquito strategies to combat the dengue menace. The present study is an extension of our earlier study aimed at determining the *in vitro* and *in vivo* protective effects of a plant-derived phytopharmaceutical drug for the treatment of dengue. In our previous report, we had identified a methanolic extract of aerial parts of *Cissampelos pareira* to exhibit *in vitro* and *in vivo* anti-dengue activity against all the four DENV serotypes. The dried aerial parts of *C. pareira* supplied by local vendors were often found to be mixed with aerial parts of another plant of the same Menispermaceae family, *Cocculus hirsutus*, which shares common homology with *C. pareira*. In the current study, we have found *C. hirsutus* to have more potent anti-dengue activity as compared with *C. pareira*. The stem part of *C. hirsutus* was found to be more potent (∼25 times) than the aerial part (stem and leaf) irrespective of the extraction solvent used, *viz*., denatured spirit, hydro-alcohol (50:50), and aqueous. Moreover, the anti-dengue activity of stem extract in all the solvents was comparable. Hence, an aqueous extract of the stem of *C. hirsutus* (AQCH) was selected due to greater regulatory compliance. Five chemical markers, *viz.*, Sinococuline, 20-Hydroxyecdysone, Makisterone-A, Magnoflorine, and Coniferyl alcohol, were identified in fingerprinting analysis. In a test of primary dengue infection in the AG129 mice model, AQCH extract at 25 mg/kg body weight exhibited protection when administered four and three times a day. The AQCH was also protective in the secondary DENV-infected AG129 mice model at 25 mg/kg/dose when administered four and three times a day. Additionally, the AQCH extract reduced serum viremia and small intestinal pathologies, *viz*., viral load, pro-inflammatory cytokines, and vascular leakage. Based on these findings, we have undertaken the potential preclinical development of *C. hirsutus*-based phytopharmaceutical, which could be studied further for its clinical development for treating dengue.

## Introduction

Dengue is a mosquito-borne disease caused by infection of one or more of the four antigenically distinct dengue virus (DENV) serotypes. The DENV is a positive single-stranded RNA virus, which belongs to the genus *Flavivirus* of the Flaviviridae family. Dengue infection has the potential of causing a pandemic and outbreaks in many parts of the world^1^. Climate change, population growth, increased international travel, rapid urbanization, and ineffectual vector control strategies have led to an expansion of the dengue disease footprint worldwide. At this time, dengue is endemic in more than 100 countries in South-East Asia, Eastern Mediterranean, Western Pacific, Americas, and Africa (Gubler, 2011; Struchiner et al., 2015; Wilder-Smith et al., 2019).

A recent study has revealed that around 390 million dengue infection cases take place per year, out of which 96 million cases show mild to severe clinical manifestations of disease (Bhatt et al., 2013). Another study revealed that around 3.9 billion people are at risk of contracting dengue disease, making it a serious global health concern (Brady et al., 2012). Asia alone is saddled with 75% of the global dengue burden (Bhatt et al., 2013), and India is hyperendemic for dengue, with a whopping 34% contribution to the global burden (Chakravarti et al., 2012; Bhatt et al., 2013; Wilder-Smith and Rupali, 2019; Wilder-Smith et al., 2019). It has been estimated that 49% of India’s population has already been infected with the DENV, although the prevalence varies among different regions (Murhekar et al., 2019). According to a Global Burden of Disease study, dengue alone inflicts a total of ∼58 million symptomatic dengue virus infections worldwide, which estimates USD 8–9 billion annual economic burden for dengue illness globally as per 2013 prices (Shepard et al., 2016; Stanaway et al., 2016).

Dengue is transmitted through the bite of infected female mosquitoes, primarily *Aedes aegypti*. The disease manifests in febrile illness, and symptoms persist for 2–7 days post 4–10 days of incubation after the mosquito bite (WHO, 2009; Wilder-Smith et al., 2019). Symptomatic dengue infection can range from mild dengue fever (DF) to severe life-threatening dengue hemorrhagic fever (DHF) and dengue shock syndrome (DSS). DF is considered to be a mild disease associated with fever along with other symptoms such as frontal headache, retro-orbital pain, muscle and joint pain, nausea, vomiting, swollen glands, or rash. Severe dengue is more threatening and involves plasma leakage, respiratory distress, fluid accumulation, severe bleeding, and organ deterioration (WHO, 2009; Muller et al., 2017; Wilder-Smith et al., 2019).

There is no proven treatment available for dengue, and patients are provided only supportive medical care, especially fluid management (WHO, 2009). DENV has four antigenically distinct serotypes, all of which co-circulate in more than 140 countries of the world (Shepard et al., 2016). The recent launch of a dengue vaccine, Dengvaxia, by Sanofi Pasteur is of limited use due to its possible disease enhancement potential in seronegative Vaccinees (Aguiar et al., 2016; Thomas and Yoon, 2019). The other two promising vaccine candidates, developed by the National Institutes of Health/Butantan/Merck and Takeda, are in the advanced phase of clinical trials (Perkel, 2016; Biswal et al., 2020; Deng et al., 2020).

Several chemical entities have been tested for anti-DENV activities, but none have shown promise to date (Beesetti et al., 2016; Dighe et al., 2019; Lim, 2019). Several repurposed drugs, such as chloroquine (Tricou et al., 2010), celgosivir (Low et al., 2014), lovastatin (Whitehorn et al., 2016), balapiravir (Nguyen et al., 2013), and prednisolone (Tam et al., 2012), have also been evaluated for their efficacy in the clinical trials. All these trials failed to meet the efficacy endpoints. No conventional drug discovery effort has yet produced any effective and safe drug for the treatment of dengue (Dighe et al., 2019). Plant-based drug discovery has also been a focus of drug development against dengue. *Azadirachta indica* (Parida et al., 2002), *Hippophae rhamnoide* (Jain et al., 2008), *Carica papaya* (Kasture et al., 2016), *Cissampelos pareira* (Sood et al., 2015), etc. are some of the plants that are traditionally used in different regions of India by tribals, traditional healers, and local people for treating symptomatically dengue-like infections (Singh and Rawat, 2017). These herbal formulations, if validated and proved for their efficacy scientifically, can provide a safe and effective treatment for dengue.

In our previous study, we identified *Cissampelos pareira* plant for its anti-dengue activity against all four DENV serotypes (Sood et al., 2015). The dried aerial parts of *C. pareira* obtained from a local vendor are frequently found to be mixed with an identical plant *Cocculus hirsutus*, as both are twining woody climbing plants belonging to the same order Ranunculales and family, i.e., Menispermaceae. Thus, evaluation of the anti-dengue activity of *C. hirsutus* was taken up. In the current study, we have focused on investigating the effects of *C. hirsutus* in *in vitro* and *in vivo* tests of protection against dengue.

We found *C. hirsutus* to have more potent anti-dengue activity as compared with *C. pareira*. This traditional plant has been used as a detoxifier, aphrodisiac, antipyretic, cardiotonic, etc., reflecting its wide medicinal utility (Marya and Bothara, 2011). Prior reports have also suggested its use for the treatment of chronic rheumatism, syphilitic cachexia, skin diseases, constipation, kidney problems, etc. (Marya and Bothara, 2011). In an animal toxicity study, aqueous extract of aerial parts of *C. hirsutus* was found to be safe, and acute toxicity was established to be higher than 3,000 mg/kg body weight (Ganapaty et al., 2002).

We report for the first time the potential of *C. hirsutus*-based drug for the treatment of dengue. We have investigated the efficacy of *C. hirsutus* extracts using different parts of plant and solvents under varied drying conditions. The anti-dengue activity was established through a flow-cytometry-based neutralization test (FNT), which is more quantitative and robust as compared with plaque-based virus neutralization assays (PRNT) (Kraus et al., 2007). Purified aqueous extract of the stem of *C. hirsutus* (AQCH) was found to possess the highest pan-DENV inhibitory activity. Through chemical fingerprinting analysis, five marker compounds, Sinococuline, Magnoflorine, 20-Hydroxyecdysone, Makisterone-A, and Coniferyl alcohol, were identified to be present consistently in different batches of AQCH. Only Sinococuline has been found to exhibit potent pan-anti-dengue activity. Furthermore, AQCH phytopharmaceutical substance was found to be protective in the primary and secondary DENV infection in AG129 mice. Importantly, AQCH phytopharmaceutical reduced serum viremia and small intestinal pathologies, *viz*., viral load, pro-inflammatory cytokines, and vascular leakage. These results have provided the optimism to advance the evaluation of AQCH as a potential phytopharmaceutical for the treatment of dengue through appropriate animal toxicity, human safety, pharmacologic, and efficacy trials.

## Materials and Methods

### Animal Ethics Statement

This study involved experiments on AG129 mice, which were performed at the International Centre for Genetic Engineering and Biotechnology, New Delhi (ICGEB/IAEC/08/2016/RGP-15), in compliance with the Committee for the Purpose of Control and Supervision of Experiments on Animals guidelines issued by the Government of India.

### Cells and Viruses for *in vitro* Dengue Virus Inhibition Assay

The Vero cell line was purchased from the American Type Cell Culture, VA, United States. This monkey kidney cell line was maintained using Dulbecco’s Modified Eagle medium (DMEM) supplemented with 10% ΔFBS in a 10% CO_2_ humidified incubator at 37°C. World Health Organization reference DENV strains DENV-1 (WP 74), DENV-2 (S16803), DENV-3 (CH53489), and DENV-4 (TVP-360) were received from Dr. Aravinda de Silva’s lab, University of North Carolina, United States. Anti-dengue 2H2 antibody was produced in-house from its hybridoma.

### Chemicals and Reference Compounds for High-Performance Liquid Chromatography

Organic solvents used in the plant extraction and high-performance liquid chromatography (HPLC) analysis were procured from E. Merck Ltd., Mumbai, India, either of analytical or HPLC grade. Before use, solvents were also filtered through a 0.45-μm membrane filter (Millipore, Billerica, MA, United States). The HPLC column RP18e Purospher-STAR (Hibar) (250 × 4.6 mm; 5μm) was used for chemical fingerprinting (E. Merck) and Eclipse 5 μm; 9.4 × 250 mm was used for purification of marker compounds (Agilent). The chemicals and reagents used for standardization and quality control were procured from Sigma-Aldrich, United States. Water for extraction and HPLC analysis was obtained from a high-purity Milli-Q Advantage A10 water purification system (Millipore, Molsheim, France).

### Plant Procurement, Validation, and Extract Preparation

The botanical raw material (BRM), i.e., aerial or stem parts of *C. pareira* and *C. hirsutus*, were collected by the botanist from Mandawara block, Lalitpur district, Uttar Pradesh, India. Identification of the collected BRM was performed at the Plant Science Division of Council of Scientific and Industrial Research—Indian Institute of Integrative Medicine (CSIR-IIIM), Jammu, India. Duly identified herbarium specimens of *C. pareira* (accession no. RRLH-23148) and *C. hirsutus* (accession no. RRLH-23152) were submitted to internationally recognized Janaki Ammal Herbarium at CSIR-IIIM, Jammu. Furthermore, for some studies, BRM of *C. hirsutus* collected from the same area was procured from the local vendor at Lalitpur. After critical macroscopic and microscopic examination, the botanical identity of the procured BRM samples of *C. hirsutus* was confirmed at the Plant Science Division of CSIR-IIIM. The duly identified samples of the procured BRM, i.e., aerial (accession nos. CDR-4037 and CDR-4038) and stem (accession nos. CDR-4061, CDR-4064, CDR-4065, and CDR-4078) parts of *C. hirsutus*, have been submitted to the Crude Drug Repository at CSIR-IIIM, Jammu.

BRM was dried in the shade and pulverized. The grounded plant masses of *C. pareira* and/or *C. hirsutus* were separately charged into an extractor at ambient temperature. The extracting solvent used were: (1) Hydro-alcohol, 50:50, (2) Denatured Spirit, and (3) Water. The extraction solvent was added to the pulverized BRM, and the mixture was heated to reflux temperature for about 3 h. The extracted mass was filtered, collected, and stored in a container. The extraction process was repeated from the residue plant mass two more times. All the three filtered extracts were combined, concentrated, and further dried either using rotor vapor, tray under vacuum, or spray drying. The yield of dry extract (drug substance) obtained was 5–14%, depending on the part of the plant used and the extracting solvent.

The phytopharmaceutical drug-based AQCH tablets 100/300/500 mg has been developed as an immediate-release formulation. The dry granulation method has been selected as the manufacturing process based on the hygroscopic nature of AQCH. The stability of AQCH tablets was extensively evaluated, and the drug product was found to be satisfactory for tested up to 6 months in accelerated conditions [40 ± 2°C/75 ± 5% relative humidity (RH) and for tested up to 18 months in long-term conditions (30 ± 2°C/65 ± 5% RH)] (Supplementary Table 3).

### Flow Cytometry-Based Virus Inhibition Assay

Vero cells were seeded in a 96-well plate (∼25,000 cells/well) in 200-μl DMEM + 10% ΔFBS and incubated for 24 h in an incubator adjusted at 37°C and 10% CO_2_. Next day, cells were infected with DENV-1, -2, -3, and -4 at a multiplicity of infection of 0.1 (5,000 fluorescence-activated cell sorting infectious unit; FIU) in 100-μl DMEM + 0.5% ΔFBS. After a 2-h incubation of Vero cells with the virus at 37°C and 10% CO_2_, virus infection media was aspirated, and a suitable range of extract/drug concentration to be tested was added to the wells in 200-μl DMEM + 0.5% ΔFBS in duplicates. Cells were incubated further for another 46 h in an incubator at 37°C and 10% CO_2_. Wells infected with the virus without any subsequent treatment served as virus controls, whereas wells with no infection and no treatment served as cell controls. These experimental controls were utilized for relative virus infection calculations and antibody background signal adjustments, respectively. Post-incubation, cells were trypsinized, fixed using 4% paraformaldehyde, and stained with anti-prM Alexa-488-labeled 2H2 antibody as reported elsewhere (Kraus et al., 2007; Shukla et al., 2020b). Stained cells were assessed for virus infection using a flow cytometer (BD FACS Verse/Lyric), and data were analyzed through Flowjo software. Extract/drug concentration resulting in a 50% reduction in the number of DENV infected cells with reference to the virus control, which represents 100% infection, was defined as the 50% inhibitory concentration (IC_50_) of the herbal extract/drug tested. All the extracts/drug/compounds prepared during this study were analyzed using this flow cytometry-based virus inhibition assay for assessing their anti-dengue potential.

### 3-(4, 5-Dimethylthiazolyl-2)-2,5-Diphenyltetrazolium Bromide Assay

The *in vitro* cell cytotoxic index (CC_50_) of AQCH was evaluated through 3-(4, 5-dimethylthiazolyl-2)-2,5-diphenyltetrazolium bromide (MTT) assay. Vero cells were seeded as described in the flow-cytometry-based virus inhibition assay. Post-24-h incubation at 37°C and 10% CO_2_, overlay media was removed, and 200 μl of a suitable concentration range of AQCH prepared in dilution media (DMEM + 0.5% ΔFBS) was added to the wells in duplicates; cells incubated with dilution media alone were kept as cell control and processed in parallel. Cells were incubated further for another 46 h in an incubator at 37°C, 10% CO_2_. Post-incubation, 10 μl of 5 mg/ml MTT reagent (procured from Sigma Aldrich, United States) prepared in PBS was added and further incubated for 2 h at 37°C, 10% CO_2_. Upon the formation of formazan crystals, the overlay was removed, and 100 μl of dimethyl sulfoxide was added. After the dissolution of crystals in dimethyl sulfoxide, absorbance was taken at 570 nm. The % cell cytotoxicity was calculated for each AQCH concentration with respect to cell control. The concentration of AQCH at which 50% cell cytotoxicity was observed is reported as CC_50_.

### Aqueous Extract of the Stem of *Cocculus hirsutus* Chemical Fingerprinting Instrumentation and Characterization Methodology

All nuclear magnetic resonance (NMR) spectral data were recorded on a Bruker 400-MHz spectrometer. Chemical shifts (δ) were referenced internally to the residual solvent peak (CD3OD: ^1^H δ 3.30, ^13^C δ 49.0 ppm; CDCl^3^: ^1^H 7.26, ^13^C 77.0 ppm), and the reference point was tetramethylsilane (δH and δC: 0.00 ppm). High-resolution electrospray ionization mass spectrometry spectra were recorded on an Agilent 1100 LC-Q-TOF mass spectrometer and HRMS-6540-UHD machines. HPLC purifications were performed on Thermo Scientific Dionex UltiMate 3000 HPLC system with ultraviolet (UV) detector. Column chromatography was performed using silica gel (60–120 and 230–400 mesh); fractions were monitored by thin-layer chromatography (TLC) using pre-coated silica gel plates 60 F254 (Merck). Spots were visualized by UV light or by spraying with H_2_SO_4_-MeOH, anisaldehyde-H_2_SO_4_ reagents.

For the HPLC fingerprinting, 100 mg of AQCH (FCH 1909006) was transferred in a 20-ml volumetric flask, added approximately 10 ml of diluent and sonicated/shaken/stirred for 5–10 min to dissolve and made up the volume with diluent and mixed and filtered through a 0.45-m filter for HPLC fingerprinting. It was performed on RP18e Purospher-STAR (Hibar) (250 × 4.6 mm; 5μm) column. The mobile phase containing a buffer (0.1% formic acid in water) and acetonitrile was used at the flow rate of 0.65 ml/min at a column temperature of 30°C at 254-nm wavelength. The volume of injection was 5 μl, and the total run time of the assay was 75 min. A gradient program was used as follows: 0–15 min, 00–05% B; 15–40 min, 05–20% B; 40–55 min, 20–30% B; 55–65 min, 30–60% B; 65–68 min, 60–00% B and 68–75 min, 00% B. For the isolation, a portion of AQCH (500 gm) was suspended in distilled water and partitioned between ethyl acetate (A) and H_2_O (B). The aqueous layer (B) was basified with NH_4_OH solution (pH 9) and then extracted with chloroform, which resulted in a chloroform layer (4.0 g, C) and aqueous layer (D). The CHCl_3_ layer (C) was further purified through repeated column chromatography in neutral alumina eluted with a gradient of CHCl_3_-MeOH (100:0–0:100) to obtain Sinococuline (1) as a major constituent along with Magnoflorine (2). The aqueous layer (D) was lyophilized (480.5 g) and suspended in methanol. The methanol soluble portion (400.0 g) was purified by column chromatography (silica gel, 100–200 mesh), eluted with a gradient of CHCl_3_-MeOH (100:0–0:100, 500-ml collected volumes of each fraction), and concentrated, giving 50 fractions (Fr.1–Fr.50), and their composition was monitored by TLC, with those showing similar TLC profiles grouped into six major fractions (Fr-1a to Fr-6a). Fraction Fr-2a afforded two UV active compounds as crystals. These crystals containing two UV active compounds were further subjected to semi-preparative C18 reversed-phase HPLC [Eclipse 5 μm; 9.4 × 250 mm; 3 ml/min; gradient of water (B)/acetonitrile (A) over 32 min; 100–80% B (5 min), 80–60% B (5 min), 60–50% B (5 min), 50–40% B (5 min), 40–20% B (5 min), 20–45% B (3 min), 45–70% B (2 min), and 70–100% B (2 min); column oven temperature 25°C] to give 20-Hydroxyecdysone (3) and Makisterone-A (4). The EtOAc soluble fraction (A) was subjected to column chromatography (silica gel, 100–200 mesh), eluted with a gradient of CHCl_3_-MeOH (100:0–0:100, 250-ml collected volumes of each fraction) and concentrated, giving 30 fractions (Fr.51–Fr.80). Coniferyl alcohol (5) was obtained from fractions Fr. 66–Fr. 70 in pure. All the isolated compounds 1–5 were identified by detailed spectral analysis using one- and two-dimensional NMR and HRESI-MS data (Supplementary Figure 1) and compared with the reported spectral data (Ishii et al., 1984; Itokawa et al., 1987; Nakasone et al., 1996; Snogan et al., 2007; Taha-Salaime et al., 2019).

### Stability Assay

AQCH was formulated into tablets of different strengths (100, 300, and 500 mg) using the approved excipients. The accelerated and long-term stability of AQCH and AQCH tablets was assessed by exposing them to different conditions (30 ± 2°C/65 ± 5% RH and 40 ± 2°C/75 ± 5% RH). The *in vitro* anti-dengue activity and the content of a selected marker compound were evaluated for the stored samples at different time points (1, 2, 3, and 6 months).

### 4G2 Antibody, DENV-2 S221, and AG129 Mice

A monoclonal anti-flavivirus antibody, 4G2, was produced in-house using hybridoma procured from the American Type Cell Culture. DENV-2 strain S221 used for *in vivo* studies was obtained from Global Vaccines Inc., NC, United States, and propagated using DMEM adapted C6/36 cell line for preparing virus stocks. The titer of these stocks was estimated using Vero cells in FIU per milliliter, as described elsewhere (Lambeth et al., 2005). AG129 mice were purchased from B&K Universal, United Kingdom, and bred in-house at the International Centre for Genetic Engineering and Biotechnology, New Delhi, for all animal experiments.

### *In vivo* Evaluation of the Efficacy of Aqueous Extract of the Stem of *Cocculus hirsutus* in AG129 Primary Dengue Lethal Mouse Model

Primary DENV infection was established in a type-I and type-II interferon (IFN) receptor knockout, AG129 mice (Shresta et al., 2006; Sarathy et al., 2015) by challenging them with a lethal dose (1 × 10^5^ FIU; intravenous) of DENV-2 S221. Mice were orally gavaged with AQCH, 33 and 100 mg/kg/day (prepared in 0.1% w/v methylcellulose), for 5 days using twice a day (BID), three times a day (TID), and four times a day (QID) dosing regimens starting 30 min post-infection as reported elsewhere (Watanabe et al., 2012). The level of protection conferred by AQCH was monitored through mortality and morbidity scores for 15 days post-lethal challenge. The morbidity score was assessed based on a five-point system: 0.5, mild ruffled fur; 1.0, ruffled fur; 1.5, compromised eyes; 2, compromised eyes with hunched back; 2.5, loose stools; 3.0, limited movement; 3.5, no movement/hind leg paralysis; 4.0, euthanized if the cumulative score was 4; 5.0, assigned to the animal that was dead as reported previously (Ramasamy et al., 2018; Shukla et al., 2020c). The level of protection was scored through % survival for 15 days post-lethal challenge.

### Aqueous Extract of the Stem of *Cocculus hirsutus* Efficacy Evaluations in Secondary Dengue Disease AG129 Mouse Model

The secondary dengue AG129 mouse model was established through intravenous inoculation of immune complexes (IC) of sublethal dose of DENV-2 S221 (2 × 10^4^ FIU) made with neutralizing concentration of 4G2 mAb (10 μg) reported elsewhere (Watanabe et al., 2015; Shukla et al., 2020c, b). IC challenged mice (*n* = 6) were orally gavaged with different doses of AQCH, 33 and 100 mg/kg/day (prepared in 0.1% w/v methylcellulose), in the BID, TID, and QID dosing regimens for 5 days post-ICs inoculation. The level of protection conferred by AQCH was examined in a similar manner as in the primary dengue AG129 model.

### Evaluation of Serum Viremia and Small Intestinal Pathologies—Viral Load, Vascular Leakage, and Pro-inflammatory Cytokine Levels in Secondary Dengue Infection AG129 Mice

Two groups of IC injected AG129 mice (*n* = 6) were orally fed with AQCH at 8.25 and 25 mg/kg/dose QID (equivalent to 33 and 100 mg/kg/day, respectively) for 4 days. Serum was collected from each group (*n* = 3) on day 4 for assessing viral RNA equivalents through real-time quantitative polymerase chain reaction, and post-sera collection vascular leakage was quantified in these mice using Evans blue dye, as detailed in the Supplementary Protocol 1. The remaining three mice from each group were killed on day 4 and small intestines harvested for assessing viral load and levels of tumor necrosis factor-alpha (TNF-α) and interleukin-6 (IL-6) cytokines as elaborated in the Supplementary Material.

### Statistical Analysis

Statistical differences between two groups of treated animals were calculated through two-way analysis of variance (with Bonferroni’s correction for multiple comparisons). The Kaplan–Meier survival curves were analyzed by the log-rank test for significance. *p*-values of ≤0.05 and <0.001 were considered significant and very significant, respectively. All statistical calculations were performed using GraphPad Prism (v8.0) software.

## Results

### Selection of *Cocculus hirsutus* for the Evaluation of Anti-dengue Activity

Guided by the Indian Ayurveda literature and our *in vitro* and *in vivo* bioassays, we had earlier identified and established that methanolic extract of the aerial parts of *C. pareira* possesses pan-anti-dengue activity (Sood et al., 2015). *C. pareira* belongs to the family Menispermaceae, which is historically known to be rich in a variety of alkaloids (Barbosa-Filho et al., 2000). In our previous study (Sood et al., 2015), a total of 19 plants were evaluated for their anti-dengue activity, two of which *C. pareira* and *Tinospora cordifolia* belonged to the family Menispermaceae, and both of them were found to possess anti-dengue activity. However, *C. pareira*, which belongs to the Cocculeae tribe of Menispermaceae, exhibited significantly stronger anti-dengue activity than *T. cordifolia*, which belongs to the Tinosporeae tribe.

In the present study, we have investigated the anti-dengue activity of plant extracts from aerial parts and stem from *C. hirsutus*, which has a morphologic resemblance to *C. pareira* but was found to be microscopically altered in their anatomical characters (Supplementary Table 1). We initiated work on *C. hirsutus* because of the finding that it is often present with *C. pareira* in dried collections made from a local vendor.

### *Cocculus hirsutus* Possesses Pan-Anti-dengue Activity and Is More Potent Than *Cissampelos pareira*

With methanolic extract of aerial parts of *C. pareira* as our benchmark, we prepared three batches each of methanolic extract of aerial parts of *C. pareira* and *C. hirsutus* in a manner similar to that stated in Sood et al. (2015). In this study, we evaluated all these six methanolic extracts for anti-dengue activity in an *in vitro* FNT instead of plaque-based bioassay used in Sood et al. (2015). The FNT and plaque-based bioassays are principally similar. However, FNT-based evaluation is advantageous because it is high-throughput and is more stringent, as it uses a higher dose of DENV for the evaluation of the anti-dengue activity. In the FNT assay used in the current study, the Vero cells were infected with 0.1 multiplicity of infection of each DENV serotypes, and post-infection the cells were incubated in media containing extract at various concentrations for 46 h. Post-incubation cells were fixed, permeabilized, and stained with Alexa fluor-labeled anti-dengue mAb, 2H2, reactive to all the four DENV serotypes, which were read in a flow cytometer to determine the percentage of DENV infected cells. This was used to calculate the extract concentration at which 50% of the DENV infection was inhibited (IC_50_). Upon parallel evaluation of all the six extracts in FNT, it was observed that all the three batches of methanolic aerial *C. hirsutus* extracts possessed significantly stronger anti-dengue activity against all the four DENV serotypes as compared with methanolic aerial *C. pareira* extracts (Supplementary Figure 1).

### Selection of Aqueous Extract of Stem of *Cocculus hirsutus* for Further Evaluation

**FIGURE 1:**
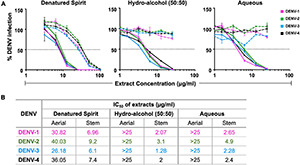
Evaluation of extracts of aerial and stem parts of *C. hirsutus* prepared using various extraction solvents: extracts of aerial and stem only parts of *C. hirsutus* were prepared in different solvents, *viz*., denatured spirit, hydro-alcohol (50:50), and aqueous. Anti-dengue activity of each of these extracts at various concentrations was evaluated against DENV-1 (magenta curve), DENV-2 (green curve), DENV-3 (blue curve), and DENV-4 (black curve) by flow-cytometry-based virus inhibition assay. **(A)** % Dengue virus (DENV) infection relative to virus control achieved is represented graphically for denatured spirit (left panel), hydro-alcohol, 50:50 (middle panel), and aqueous (right panel) aerial (dashed curves) and stem (solid curves) extracts. **(B)** Concentration of extract (μg/ml) that resulted in 50% inhibition of viral infection as compared with virus control [represented by horizontal dotted line in panel **(A)**], calculated as IC_50_ using Graphpad Prism, is shown in table for all extracts.

Upon finding *C. hirsutus* possessing more potent anti-dengue activity as compared with *C. pareira*, we explored individual preparation of extracts of both aerial (Figure 1A; dashed curves) and stem portions (Figure 1A; solid curves) of *C. hirsutus* in various solvents such as denatured spirit, hydro-alcohol (50:50), and water. These extracts were evaluated against all the four DENV serotypes by FNT, and their IC_50_ values were compared (Figure 1B). It was observed that the stem portion of *C. hirsutus* was significantly more potent than the aerial part, irrespective of the solvent used. Thus, aqueous extract of stem of *C. hirsutus*, hereafter referred to as AQCH, was advanced further due to regulatory compliance factors.

### Characterization of Aqueous Extract of the Stem of *Cocculus hirsutus* and Identification of Chemical Markers

**FIGURE 2:**
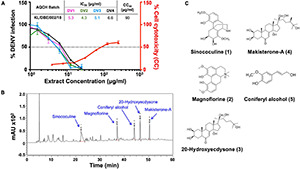
Chemical fingerprinting of AQCH: **(A)** AQCH batch KL/DBE/002/18 was prepared, and its anti-dengue activity against DENV-1 (magenta curve), DENV-2 (green curve), DENV-3 (blue curve), and DENV-4 (black curve) was confirmed by flow-cytometry-based virus inhibition assay as represented by graph of % DENV infection on left *y*-axis and extract concentration. Extent of cell cytotoxicity caused by AQCH (represented by red curve) was also measured by MTT assay that is reflected on right *y*-axis of graph as % cell cytotoxicity for given extract concentrations on *x*-axis. CC_50_ and IC_50_ values corresponding to concentration of AQCH that is toxic for 50% of cells and at which 50% of DENV infection is inhibited as compared with virus control, respectively, has been represented by a dotted horizontal line; a table of IC_50_ and CC_50_ values has been provided as an inset. **(B)** High-performance liquid chromatography (HPLC) chemical fingerprinting profile of AQCH with peaks corresponding to five identified marker compounds annotated. **(C)** Chemical structure of five marker compounds (1–5).

High-performance liquid chromatography (HPLC) was performed on the AQCH batch (ID: KL/DBE/002/18) post-confirmation of its pan-anti-dengue activity by FNT (Figure 2A) for its chemical profiling. Additionally, the extent of possible *in vitro* cytotoxicity caused to Vero cells by AQCH was also evaluated by MTT assay, and the CC_50_ was determined to be ∼90 μg/ml (Figure 2A). The HPLC chromatogram obtained is shown in Figure 2B. This was followed by isolation of five marker compounds using repeated chromatographic methods and characterized using advanced one- and two-dimensional NMR spectroscopic and mass analysis. Marker compounds were identified to be Sinococuline (1), Magnoflorine (2), 20-Hydroxyecdysone (3), Makisterone-A (4), and Coniferyl alcohol (5) (Figure 2C). The physicochemical data of all the five identified marker compounds are given in Supplementary Table 2.

**FIGURE 3:**
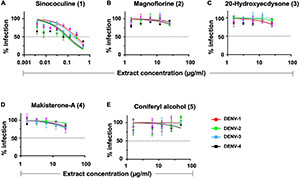
*In vitro* flow-cytometry-based dengue virus (DENV) inhibition analysis of all AQCH marker compounds. Five makers compounds **(A–E)** isolated from AQCH were tested for their anti-dengue inhibitory potency against DENV-1 (magenta curve), DENV-2 (green curve), DENV-3 (blue curve), and DENV-4 (black curve) serotypes using *in vitro* flow-cytometry-based virus inhibition assay. *X*-axis denotes compound concentrations (μg/ml) in each panel, and *y*-axis represents percent virus inhibition against virus control (no compound was added). Horizontal dotted line of each panels shows 50% inhibition of viral infection as compared with virus control, and IC_50_ was calculated for each of compounds using Graphpad Prism.

The robustness and consistency of AQCH were monitored by the preparation of various batches of AQCH extract utilizing one of the three drying methods—rotar vapor drying, vacuum tray drying, and spray drying. Irrespective of the method used for drying, the *in vitro* anti-dengue activity of all the extract batches prepared was comparable (Supplementary Figure 2A). The HPLC chromatograms of three batches corresponding to the three drying methods were observed to be overlapping (Supplementary Figure 2B), with a high degree of consistency in retention times of the five marker compounds (Supplementary Figure 2C). This indicates that the AQCH extract preparation method is consistent and robust, and the choice of drying method does not have any implication on its chemical profiling and biological activity. Thus, spray drying was considered as the method of choice, as it resulted in the formation of free-flowing finer extract in a shorter span of time, which is industrially more compatible. Furthermore, the identified compound markers were assessed for their pan anti-dengue inhibitory potency by FNT (Figure 3), and only Sinococuline was found to have anti-dengue inhibitory activity against all four DENV serotypes (Figure 3A). The other chemical entities characterized in the drug substance, Magnoflorine, 20-Hydroxyecdysone, Makisterone-A, and Coniferyl alcohol, showed almost negligible anti-dengue activity (Figures 3B–E).

### *In vivo* Evaluation of Aqueous Extract of the Stem of *Cocculus hirsutus* for Anti-dengue Activity in Primary Dengue Disease AG129 Mouse Model

**FIGURE 4:**
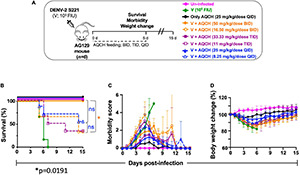
Effect of AQCH dose and dosing regimen against lethal primary DENV infection. **(A)** Schematic representation of study design. Groups of AG129 mice (*n* = 6) inoculated with lethal dose of mouse-adapted DENV-2 S221 virus (10^5^ FIU/Mouse); thereafter, mice were orally gavage with two doses of AQCH, *viz*., 100 mg/kg/day (solid curves) and 33 mg/kg/day (dashed curves) in three different dosing regimen, i.e., twice a day (BID; orange curves), thrice a day (TID; purple curves), and four times a day (QID; blue curves) for 5 days and mice were monitored for another 10 days, for their survival **(B)**, morbidity **(C)**, and body weight change **(D)**. Uninfected (neither infected with virus nor fed with AQCH; represented by magenta), virus-infected, V (infected with virus but not fed with AQCH; represented by green curves), and Only AQCH (not infected with virus but fed with AQCH at 25 mg/kg QID; black curves) groups of AG129 mice served as experimental controls. Not significant (ns) and single star (*) of right side of survival graph denotes their statistical differences between same dosing schedule of different doses.

To assess the *in vivo* AQCH efficacy, we tested AQCH in dengue-sensitive AG129 mouse model. AG129 mice are IFN type I and II receptor-deficient mice that allow viral infection to propagate and develop dengue viral symptoms. To develop the DENV disease symptoms in AG129 mice, we optimized the mouse-adapted DENV-2 strain S221 dose, which could develop dengue severe disease symptoms in the absence of any antiviral treatment. Briefly, AG129 mice (*n* = 6) were challenged with a lethal dose (1.0 × 10^5^ FIU) of DENV-2 S221, which killed all the mice by days 6–7. We utilized this primary DENV infection model for the assessment of AQCH efficacy *in vivo*. In this experiment, we tested AQCH in three different dosing regimens, i.e., BID (orange curves), TID (purple curves), and QID (blue curve) in Figure 4, at two different doses of 100 mg/kg/day (solid curves) and 33 mg/kg/day (dashed curves) (Figure 4A). It was observed that BID and TID regimens too conferred similar protection, 100% survival at 100 and ∼33% at 33 mg/kg/day, as fed QID for 5 days post-infection. Thus, there was no significant difference in the survival scores of mice between different dosing regimens at either of the two AQCH doses (*p* > 0.9999). However, the morbidity and weight loss scores of mice were moderately higher under the BID regimen (*p* = 0.0019) than TID (*p* = 0.0295) and QID for each of the two doses (Figures 4C,D). Outcomes with control mice groups were observed as expected as 0% survival in the unfed infected group “V” (green curve) and 100% survival in AQCH-fed uninfected group “Only AQCH” (black curve). Among the two doses, significantly lower protection and higher morbidity were observed at 33 mg/kg/day AQCH treatment in the BID and TID dosing regimens (*p* = 0.01).

### *In vivo* Evaluation of Aqueous Extract of the Stem of *Cocculus hirsutus* for Anti-dengue Activity in Secondary Dengue AG129 Mouse Model

**FIGURE 5:**
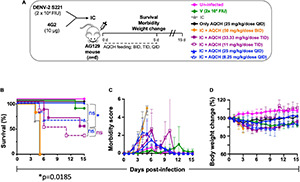
Effect of AQCH dose and dosing regimen against secondary DENV infection: **(A)** schematic representation elaborating that AG129 mice (*n* = 6) were injected with sub-lethal dose of DENV-2 S221 alone (marked as V; green curve) or as DENV-2 S221-4G2 IC (IC). IC injected AG129 mice were orally fed with either AQCH at indicated higher (solid curve) and lower (dashed curve) doses or only methylcellulose (marked as IC; gray curve) for 5 days in BID (orange), TID (purple), and QID (blue) dosing regimens. Uninfected (magenta curves) and Only AQCH (black curves) groups of AG129 mice were used as experimental controls. **(B–D)** represents their survival curves, morbidity score, and % body weight changes, respectively, which were observed during entire study duration of 15 days. Statistical differences (ns; not significant, and *; less significant) of survival between different dosing schedules are represented on the right side of graph.

Secondary dengue infection is one of the worst scenarios in dengue infection and develops very severe dengue disease through antibody-dependent enhancement (ADE) pathway (Halstead et al., 2010; Guzman et al., 2013). Thus, we investigated the therapeutic potential of AQCH against severe DENV infection using the secondary dengue AG129 mouse model. The IC of DENV-2 S221 were made *in vitro* by mixing 100% neutralizing concentration of monoclonal antibody 4G2 (10 μg). The 4G2 mAb potentially binds the fusion loop of all four DENV serotypes and tends to enhance DENV infection by following Fcγ receptor-mediated intrinsic ADE, which is manifested as severe dengue infections (DHF/DSS) as evidenced elsewhere (Halstead et al., 2010; Shukla et al., 2020a). We used this ADE model to evaluate AQCH efficacy *in vivo*. The IC inoculated mice were fed orally with previously primary dengue disease model tested doses of AQCH, *viz*., 33 (dashed curves) or 100 mg/kg/day (solid curves), using BID (orange curves), TID (purple curves), and QID (blue curves) dosing regimens for 5 days post-IC inoculation (Figure 5). The outcomes with control groups, *viz*., “Un-infected” (magenta curve), “V” (green curve), “Only AQCH” (black curve), and “IC” (gray curves), were as expected. Unlike the case in the primary dengue AG129 model, the efficacy of AQCH was not found to be similar across the three evaluated dosing regimens. QID was the most effective dosing regimen followed by TID and BID as 100%, 50%, and 0% mice survival rates were observed, respectively, with AQCH treatment at 100 mg/kg/day dose (Figure 5B). Since protection was not observed at 100 mg/kg/day AQCH dose in BID regimen, a lower AQCH dose of 33 mg/kg/day was evaluated only in TID and QID regimens, where it exhibited ∼33% and 50% protection, respectively (Figure 5B), which suggests that BID and TID dosing regimens are not appropriate regimens for treating severely infected AG129 mice. Also, morbidity scores and body weight changes correlated well across BID, TID, and QID regimens at 100 mg/kg/day with the observed survival efficacies (Figures 5C,D).

### Determining the Efficacy of Aqueous Extract of the Stem of *Cocculus hirsutus* in Preventing Dengue Disease Pathogenesis—Serum Viremia, and Small Intestinal Pathologies—Viral Load, Cytokine Storm, and Vascular Leakage in Secondary Dengue AG129 Mice Model

Severe dengue disease is known to be associated with small intestinal pathologies of enhanced viral load, increased levels of pro-inflammatory cytokines, and vascular permeability (Vejchapipat et al., 2006; Watanabe et al., 2018). Thus, it was of interest to determine the impact of AQCH treatment on small intestinal pathology, which could be examined in secondary dengue disease AG129 mice exhibiting ADE-mediated severe dengue.

**FIGURE 6:**
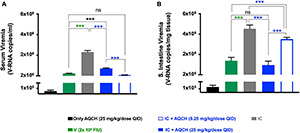
Serum and intestinal viral inhibition by AQCH in secondary DENV infection: AG129 mice (*n* = 3) inoculated with DENV-2 S221-4G2 IC to establish secondary dengue infection were orally fed with either 25 mg/kg QID (blue solid bar) or 8.25 mg/kg QID (blue empty bar) of AQCH. Serum samples were withdrawn on day 4, and mice were killed to collect small intestinal tissue. Serum viremia levels **(A)** and small intestinal viral load **(B)** were determined using SYBR green-based real-time PCR. “Only AQCH,” “V,” and “IC” control groups are shown in black, green, and gray bars, respectively. Signals detected in “Only AQCH” group (black bars) considered as real-time quantitative polymerase chain reaction background, as this group was fed with AQCH (25 mg/kg QID) only and not infected with virus. Statistical differences between groups are presented on the top of bar graphs, ns, not significant (*p* ≥ 0.05), ***very high significant difference (*p* < 0.0001).

It has been reported that small intestinal pathology is a better correlate of protection than serum viremia levels in dengue disease (Watanabe et al., 2018). Thus, we evaluated the effect of AQCH treatment on both the serum viremia and small intestinal viral load. Secondary dengue disease AG129 mice (*n* = 3) were orally fed with AQCH (8.25 and 25 mg/kg/dose, QID) for 4 days. Mice injected with IC showed symptoms of disease onset such as lethargy, whereas the mice treated with 25 mg/kg/dose QID of AQCH showed no disease symptoms (Supplementary Media File). Viremia levels were determined in serum and small intestinal tissue samples collected on day 4. Mice treatment with AQCH significantly reduced (*p* < 0.0001) the virus load in both the serum (Figure 6A) and small intestine (Figure 6B). However, unlike the observation with small intestinal viral load, reduction in serum viremia was not found to be dose-dependent, as QID treatment with 8.25 mg/kg/dose (blue empty bar) of AQCH led to a higher reduction in serum viremia than 25 mg/kg/dose, blue solid bar (Figure 6A).

**FIGURE 7:**
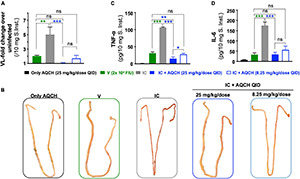
Inhibition of intestinal vascular leakage and reduction in cytokine storm by AQCH in secondary dengue virus (DENV) infection. Secondary dengue AG129 mice (*n* = 6) were treated with AQCH either at 25 mg/kg (solid blue bar) or 8.25 mg/kg (empty blue bar) QID dose for 4 days. **(A)** On day 4, three AG129 mice of each group were examined for vascular leakage in small intestine by Evans blue staining assay. Data are depicted as fold-change in dye staining with reference to an untreated control that had not received any AQCH treatment. A fold-change of 1 corresponds to basal vascular leakage equivalent to untreated group. Remaining three mice of each group were killed on day 4, perfused them with sterile PBS, and luminal content was flushed again with PBS for identification of visual intestinal vascular leakage, a representative vascular leak from each group is shown in panel **(B)**. Fifty milligrams of small intestines from panel **(B)** were prepared for determination of TNF-α **(C)** and IL-6 **(D)** by enzyme-linked immunosorbent assay, as reported earlier (Shukla et al., 2018). Control groups used in this study were “Only AQCH” group (black bar) and Virus only group (V; green bar); IC group (gray bar) is shown in each of panels. Statistical differences between two groups were calculated through two analysis of variance with Bonferroni correction and shown at top of bar graphs; ns, not significant, *p = 0.01, **p = 0.001, and ***p < 0.0001.

Since vascular leakage is a prominent manifestation of severe dengue infections (DHF/DSS), we assessed intestinal vascular leakage using Evans blue dye, which serves as a marker for albumin extravasation. The leakage was quantified through colorimetric estimation of the dye amount absorbed, which was represented as a fold increase in OD_620_ per gm of tissue’s wet weight in comparison with the “Un-infected” control. AQCH treatment was observed to significantly (*p* = 0.0015) reduce the vascular leakage at both the dose levels—25 and 8.25 mg/kg/dose QID (Figure 7A, solid and empty blue bars, respectively). Enhanced vascular leakage was observed in the “IC” mice group (Figure 7A, gray bar), as expected. The visual examination of day 4 small intestines of mice also reflected similar findings with distinctive fluid accumulation in the “IC” group, whereas the small intestines of other groups—“V,” “IC + 25 mg/kg/dose AQCH QID,” and “IC + 8.25 mg/kg/dose AQCH QID”—appeared normal and similar to the “Only AQCH” group (Figure 7B).

The manifestation of severe dengue infections occurs during the critical phase of the infection, and levels of pro-inflammatory cytokines, such as TNF-α and IL-6, become elevated, which leads to further disease progression and causes DHF/DSS. To assess the anti-inflammatory activity of AQCH, we evaluated the expression levels of pro-inflammatory cytokines, TNF-α (Figure 7C), and IL-6 (Figure 7D), in small intestines of mice. The results obtained were in correlation with the findings of vascular leakage examination. Treatment with AQCH inhibited the secretion of cytokines in the small intestine in a dose-dependent manner. The “IC” group animals showed the highest expression levels of cytokines (TNF-α and IL-6), which was expected due to 4G2-mediated ADE in secondary dengue infection AG129 mice (Figures 7C,D).

### Preparation of Aqueous Extract of the Stem of *Cocculus hirsutus* Drug Product

With the finding of strong and consistent *in vitro* and *in vivo* anti-dengue potency of AQCH, we formulated the drug substance into 100-, 300-, and 500-mg strengths of AQCH tablets (drug product). The tablets were subjected to accelerated and long-term stability studies along with the AQCH drug substance. Samples from the stability study were analyzed for Magnoflorine content and *in vitro* anti-DENV-2 activity by FNT to evaluate any deterioration or loss in bioactivity upon storage under the conditions stated. It was found that there was neither a significant change in the Magnoflorine content (Supplementary Table 3) nor any loss of anti-DENV-2 activity (Table 1) of AQCH and AQCH tablets of all the three strengths (100, 300, and 500 mg) under the condition tested up to 6 months. The long-term stability study is ongoing, and samples will be evaluated for up to 3 years of storage.

**TABLE 1:** Anti-DENV-2 activity of AQCH and AQCH tablets under the stated conditions of storage.

**AQCH extract/Tablet (batch ID)**	**Storage condition**	**DENV-2 IC_50_ (μg/ml)^a^**			
		**1 Month**	**2 Month**	**3 Month**	**6 Month**
Extract	40 ± 2°C,	4.2	4.0	5.4	7.2
(FCH1901002)	75 ± 5% RH				
	30 ± 2°C,	nd*	nd*	6.1	5.7
	65 ± 5% RH				
Tablet 100 mg	40 ± 2°C,	4.7	4.6	7.3	5.4
(RYP(6665)079A)	75 ± 5% RH				
	30 ± 2°C,	nd*	nd*	7.8	4.7
	65 ± 5% RH				
Tablet 300 mg	40 ± 2°C,	5.2	6.2	6.2	5.3
(RYP(6665)079B)	75 ± 5% RH				
	30 ± 2°C,	nd*	nd*	6.1	4.0
	65 ± 5% RH				
Tablet 500 mg	40 ± 2°C,	4.6	2.7	5.1	5.1
(RYP(6665)079C)	75 ± 5% RH				
	30 ± 2°C,	nd*	nd*	6.8	4.7
	65 ± 5% RH				

*^a^Anti-DENV-2 activity is measured as IC_50_, which corresponds to the concentration at which 50% of the virus infection is inhibited with respect to virus control.*

nd*, not determined; these specific storage conditions were for long-term stability studies and were therefore not sampled on 1^st^ and 2^nd^ month of storage.

## Discussion

Plants have been known and traditionally used worldwide for their therapeutic potential since time immemorial. Their therapeutic usefulness has been documented all around the globe in various traditional pieces of literature. Hence, these classical troves, which detail the medicinal utility of plants, are an attractive repertoire of knowledge that could be explored through contemporary methods for the development of safe and effective therapies for various diseases. This has ushered the development of many plant-derived molecules, which today are in clinical use globally, morphine being the first FDA-approved plant-derived molecule (Li and Weng, 2017). Additional examples include antimalarials such as Quinine from Cinchona bark used widely. It is also noticed that approximately 25% of the drugs prescribed worldwide are derived from plants, and such active compounds are in use (Sahoo et al., 2009).

Dengue is one of the world’s rapidly spreading arboviral diseases, with the incidence of symptomatic dengue doubling every decade (Stanaway et al., 2016). The highest burden of this disease lies in Southeast Asia, with India being one of the epicenters (Bhatt et al., 2013; Stanaway et al., 2016). According to a study, actual dengue cases in India are ∼282 times higher than that reported annually, having an economic impact of USD ∼1.11 billion (Shepard et al., 2014). Thus, there is a dire need for an effective dengue vaccine and/or drug to fight against dengue. There have been many efforts toward the development of drugs for dengue, including the repurposing of approved drugs (Low et al., 2017; Botta et al., 2018). However, none of the drugs have yet succeeded in proof-of-concept trials, and thus, a dengue antiviral still remains an unmet need.

Our group had previously evaluated 19 medicinal plants for their anti-dengue activity that led to the identification of *C. pareira* of the Menispermaceae family as the most potent plant (Sood et al., 2015). Our continued exploration has led to the identification of *C. hirsutus* with significantly higher anti-dengue potential than *C. pareira.* When the methanolic extracts of aerial parts of *C. hirsutus* and *C. pareira* were compared head-on in an *in vitro* FNT assay, we found that *C. hirsutus* have significantly more potent anti-dengue activity (Supplementary Figure 1).

We next investigated if methanol extraction could be replaced with milder solvents such as denatured spirit, hydro-alcohol (50:50), or water. In a series of investigations, we prepared the extract of stem and aerial parts of *C. hirsutus* in various solvents and evaluated the drug substance for their anti-dengue activity (Figure 1). These experiments yielded two important outcomes. First, irrespective of the solvent used, the stem of *C. hirsutus* was significantly more potent in its anti-dengue activity than the aerial part. Second, the stem extract prepared in water was comparable in its anti-dengue activity to other tested solvents. Thus, aqueous extract of the stem of *C. hirsutus* (AQCH) was selected as the extract of choice for the preparation of lab-scale and GMP scale drug substances.

After establishing the anti-dengue potential of AQCH through bioassays, we further evaluated the industrial viability of AQCH drug substance production. For this, an AQCH batch was profiled through HPLC, and five marker compounds—Sinococuline, Magnoflorine, 20-Hydroxyecdysone, Makisterone-A, and Coniferyl alcohol—were identified (Figure 2). The chemical profiling data of a bioactive batch of AQCH enabled us to modify our extraction method without compromising the bioactivity or quality of the extract prepared. Thus, various batches of AQCH were prepared using three different drying methods, *viz*., rotary vapor, vacuum tray, or spray drying. All the AQCH batches were found to exhibit similar anti-dengue activity (Supplementary Figure 2A) irrespective of the drying process, indicating the robustness and consistency of the method of extraction. This was corroborated by their HPLC chemical fingerprinting that yielded similar chromatograms (Supplementary Figures 2B,C). Spray-dried AQCH extracts were utilized for further evaluations due to greater industrial compatibility. Additionally, the identified compound markers were evaluated for their pan-anti-dengue inhibitory potency by flow-cytometry based inhibition assay (Figure 3), and only Sinococuline was found to have anti-dengue inhibitory activity against all four DENV serotypes.

AQCH drug substance was evaluated for its protective efficacy *in vivo* in AG129 mice in primary (Figure 4) and secondary dengue infection (Figure 5) settings, which is an established model for the evaluation of antivirals (Watanabe et al., 2018). AG129 mice being deficient in IFN-α/β and γ receptors allow propagation of DENV and development of dengue disease-like symptoms, such as high viremia, vascular leakage, elevated pro-inflammatory cytokines, low platelets, high hematocrit, and death (Johnson and Roehrig, 1999; Chan et al., 2015). In the primary dengue AG129 infection model, mice were challenged with a lethal dose of mouse-adapted DENV-2 strain S221 and subsequently treated with AQCH. However, in the secondary dengue infection model, AG129 mice were inoculated with *in vitro* made IC of sub-lethal dose of DENV-2 S221 and neutralizing concentration of cross-neutralizing monoclonal antibody, 4G2. 4G2 essentially binds to the fusion-loop of all four DENV serotypes and tends to enhance dengue virus infection. A sub-lethal dose of DENV-2 S221 does not cause any mortality in mice; however, it becomes lethal when complexed with 4G2 mAb due to ADE and mimicking a lethal secondary dengue infection.

In both primary and secondary dengue AG129 infection models, two doses of AQCH (100 and 33 mg/kg/day) were evaluated in each of the three dosing regimens (BID, TID, and QID), where the dose-dependent protection was observed with 100 mg/kg/day being more efficacious than 33 mg/kg/day in all the three dosing regimens in both the models (Figures 4, 5). Evaluation of different dosing regimens for any new anti-dengue drug becomes crucial as the potency can be enhanced with increased dosing frequency. This was well illustrated by Celgosivir, which was found to be less effective at 100 mg/kg/day single dose than at 50, 25, or even 10 mg/kg/day given BID in AG129 mice (Watanabe et al., 2012). In this study also, increased dosing frequency yielded significantly higher survival efficacies. The effect of the dosing regimen was less pronounced in primary AG129 mice (Figure 4); however, it became more discernible in the secondary dengue infection model, where QID feeding of 100 mg/kg/day resulted in the survival of 100% of animals, whereas the BID feeding resulted in the survival of none (Figure 5). Hence, all further *in vivo* evaluations of AQCH were performed at the most protective dose and a dosing regimen of 25 mg/kg/dose QID (equivalent to 100 mg/kg/day).

The AG129 mice exhibit enhanced serum viremia and acute small intestinal pathologies during secondary DENV infection (Watanabe et al., 2015). Thus, we examined the impact of AQCH drug substance treatment in the secondary dengue AG129 model on serum viremia as well as markers of small intestinal pathology, *viz*., viral load, vascular leakage, and TNF-α and IL-6 levels. It was observed that AQCH treatment reduced both the serum and small intestinal viral loads. However, unlike small intestinal viremia, the serum viral load reduction was not dose-dependent (Figure 6). AQCH treatment at 8.25 mg/kg/dose QID (33 mg/kg/day) led to a higher reduction of serum viremia than at 100 mg/kg/day, which does not correlate with survival data (Figure 5B). This indicates that the serum viremia levels are not truly correlative of protection in coherence with Watanabe’s observations (Watanabe et al., 2015). Like viremia, cytokine expression levels and extent of vascular leakage in the small intestine too were dose-dependent with AQCH at 8.25 mg/kg/dose QID (33 mg/kg/day), offering lower reduction than 100 mg/kg/day dose (Figure 7). This corroborated well with the lower survival efficacy of secondary dengue AG129 mice upon treatment with 33 mg/kg/day (Figure 5B). These observations indicate that small intestinal pathology indeed correlates well with the protection efficacy of an anti-dengue drug.

Demonstration of potent anti-dengue activity by AQCH in *in vitro* and *in vivo* analyses lays the ground for its clinical development. We chose a batch of AQCH extract, FCH1901002, and formulated it into tablets of various strengths (100, 300, and 500 mg), which were found to be stable for more than 6 months upon storage (Table 1). This was also evidenced in *in vivo* protective efficacy and showed similar protection as AQCH at 25 mg/kg/dose QID (data not shown here). This supports the case for the potential preclinical development of AQCH to proceed further for its clinical development to combat dengue fever.

In conclusion, this is the first study reporting an aqueous extract of the stem of *C. hirsutus* to possess significant pan-anti-dengue activity; the extraction process is robust and consistent, making this plant industrially viable for further clinical development. At this time, when there is no approved anti-dengue drug available, this phytopharmaceutical formulation can be a breakthrough in providing an efficacious and economical remedy to combat dengue, especially in resource-poor nations.

## Conclusion/Significance

This work provides the first evidence of the pan-anti-dengue potential of *C. hirsutus*-based phytopharmaceutical as determined through extensive *in vitro* and *in vivo* experiments. We have also characterized five chemical entities in the AQCH phytopharmaceutical substance, which provides means for the standardization of phytopharmaceutical drug substances and phytopharmaceutical products. Based on these findings, a program to develop a safe and effective *C. hirsutus*-derived phytopharmaceutical product for the treatment of dengue has been initiated.

## Limitations

Although both the *in vitro* and *in vivo* models of dengue virus infections used in the current study have been extensively deployed for the development of a potential preclinical AQCH phytopharmaceutical against dengue virus infections, these models may not reflect the true efficacy of AQCH phytopharmaceutical in dengue-infected humans. In the absence of an ideal model system, additional studies need to be performed to establish the anti-dengue potential of AQCH for the treatment of dengue infections in humans.

## Data Availability Statement

The raw data supporting the conclusions of this article will be made available by the authors, without undue reservation.

## Ethics Statement

The animal study was reviewed and approved by the Institutional Animal Ethics Committee (IAEC) of International Centre for Genetic Engineering and Biotechnology, New Delhi (ICGEB/IAEC/08/2016/RGP-15) in compliance with the “Committee for the Purpose of Control and Supervision of Experiments on Animals (CPCSEA)” guidelines issued by the Government of India.

## Author Contributions

NK, UA, and AAL: study conceptualization. SG and PK: plant identification. KN: plant extract preparation. RS and HB: animal experiments. AP, RKR, HB, and RS: virus inhibition assays. RS, AP, and RKR: virus culture and titration. RA: serum and tissue viral estimation. RKR: MTT assay. RKR, TJ, RP, HM, and SM: tablet formulation and stability studies. DS, VS, PG, APG, YSB, and RAV: chemical fingerprinting and marker compound isolation. RS, RKR, AP, RA, HB, RKS, KN, DS, VS, YSB, RAV, TJ, BV, AAL, UA, and NK: analysis and interpretation of data. UA, RKR, RS, and NK: drafting of the manuscript. RS, RKR, AP, RA, HB, RKS, SC, KN, DS, VS, PG, APG, SG, PK, YSB, TJ, BV, RP, HM, SM, RK, RSo, RAV, DSR, AAL, UA, and NK: approval of final manuscript. All authors contributed to the article and approved the submitted version.

## Conflict of Interest

This study received funding from Sun Pharmaceutical Industries Limited, which was provided for the conduct of work to IIIM, Jammu, and ICGEB, New Delhi. The investigators from the funder had the following involvement with the study–(1) Design, conduct, analysis, and interpretation: UA, RKR, RA, HB, RSo, and AAL; (2) preparation and characterization of the drug substance and drug product: KN, TJ, BV, RP, HM, SM, and RK. RKR, RA, HB, KN, TJ, BV, RP, HM, SM, RK, RS, AAL, and UA were employed by company Sun Pharmaceutical Industries Limited. The remaining authors declare that the research was conducted in the absence of any commercial or financial relationships that could be construed as a potential conflict of interest.

## Publisher’s Note

All claims expressed in this article are solely those of the authors and do not necessarily represent those of their affiliated organizations, or those of the publisher, the editors and the reviewers. Any product that may be evaluated in this article, or claim that may be made by its manufacturer, is not guaranteed or endorsed by the publisher.

## Footnotes

1
https://www.who.int/denguecontrol/disease/en/
