# Supplementary material for: Cocculus hirsutus-Derived Phytopharmaceutical Drug Has Potent Anti-dengue Activity
Source: Front Microbiol. 2021 Nov 29;12:746110. doi: 10.3389/fmicb.2021.746110 (PMC8667597; doi:10.3389/fmicb.2021.746110)
Supplement: Supplementary file 2 [file Data_Sheet_1.docx]

***Cocculus hirsutus*-derived phytopharmaceutical drug has potent anti-dengue activity**

Shukla *et al.,*

**Supplementary Material**

**Supplementary table 1:** Comparative anatomy of stem of *Cocculus hirsutus (L.) and Cissampelospariera (L.)*

| **Anatomical criterions** | ***Cocculus hirsutus* (L.)** | ***Cissampelospariera*(L.)**. |
| --- | --- | --- |
| Epidermis | Epidermal cells are rectangular shaped. | Epidermis single layered. Outer walls of cells are cuticularised |
| Hypodermis | Single-layered thick-walled hypodermis. | 2-3 layers of collenchyma followed by 3-4 layers of chlorenchymatous hypodermis |
| Cortex | Multi-layered cortex. The cortical cells are parenchymatous and hexagonal shaped. | Cortical region is absent |
| Vascular Bundle | The vascular bundles are open, end arch, conjoint and collateral with phloem facing towards epidermis and xylem towards the pith region. Individual vascular bundles are separated from each other by vascular rays. Below the xylem of each vascular bundle, there is a patch of sclerenchyma tissue. | Sclerenchyma fibres form arcs joined by stone cells in the vascular rays making a continuous ring. Inside of the sclerenchyma, parenchyma caps are present. Open collateral 8-10 vascular bundle; adjacent vascular bundles are divided by parenchymatous |
| Pith | Prominent pith is present in the centre. | Pith is small and composed of large parenchyma cells |

**Supplementary Figure 1: *C. hirsutus* possesses a more potent anti-dengue activity than *C. pareira.* (A)** Three consecutive batches of the methanolic extracts of aerial parts of *C. pareira* and *C. hirsutus* were evaluated for their anti-dengue activity at 3.12, 6.25, 12.5, 25, 50 and 100 µg/ml extract concentrations, and the % DENV infection was recorded in a flow-cytometry-based virus inhibition assay against all the four DENV serotypes. The concentration of extract (µg/ml) that resulted in 50% inhibition of viral infection as compared to virus control was calculated as IC_50_ using Graphpad Prism. The IC_50_ values were calculated separately for each of the three extracts prepared from both the plants and their geometric mean IC_50_ values against each of the four DENV serotypes are as presented in the table. IC_50_ values of aerial methanolic *C. pareira* extract from plaque reduction neutralisation assay reported in Sood*et al.,* 2015 taken as reference for the current study are also shown in the table. Graphs **(B-E)** are the representative of the same three consecutive batches of the methanolic extracts of aerial parts of *C. pareira* (in broken lines) and *C. hirsutus* (in solid lines), where all the six extract were analysed against all four dengue serotypes, **DENV-1 (B: magenta curves),DENV-2 (C: green curves), DENV-3 (D: blue curves)** and **DENV-4 (E: black curves)** and plotted through GraphPad prism with y-axis represents % DENV infection, x-axis denotes extract concentration and dashed horizontal line shows 50% DENVs infection across all the four panels.

**
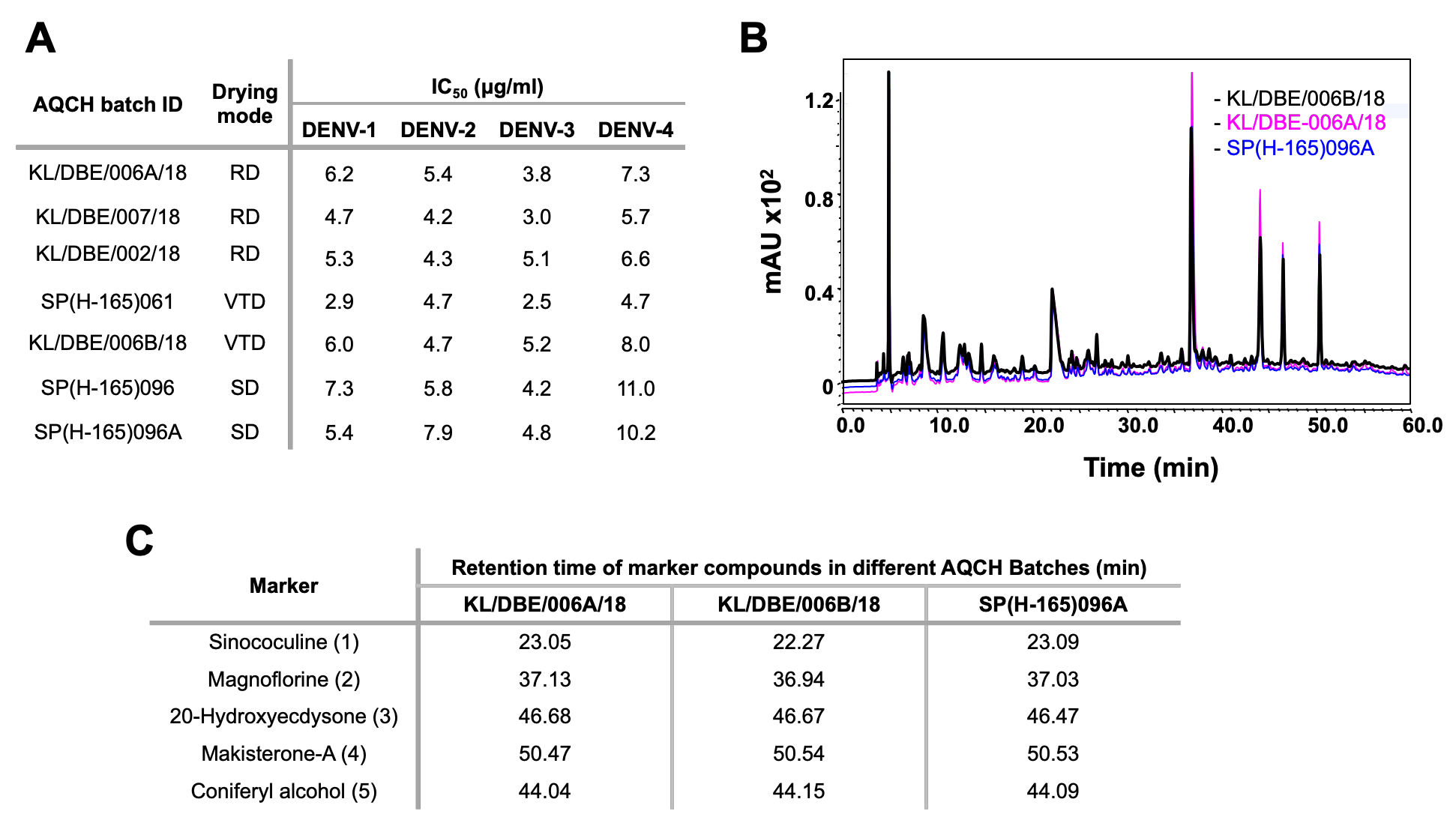
**

**Supplementary Figure 2: AQCH preparation method is consistent and robust:** Various batches of AQCH were prepared and dried through either of the three different methods viz., rotary vapour drying (RD), vacuum tray drying (VTD) or spray drying (SD). The effect of drying method was evaluated through the assessment of **(A)** anti-dengue activity by flow-cytometry-based virus inhibition assay yielding IC_50_ values (concentration of the extract required to reduce the DENV infection by 50% as compared to virus control), and **(B,C)** chemical fingerprinting profile; an overlay HPLC chromatograms of the three batches corresponding to the three drying conditions and a table of retention time of five marker compounds are shown in panels ‘**B’** and ‘**C’**, respectively.


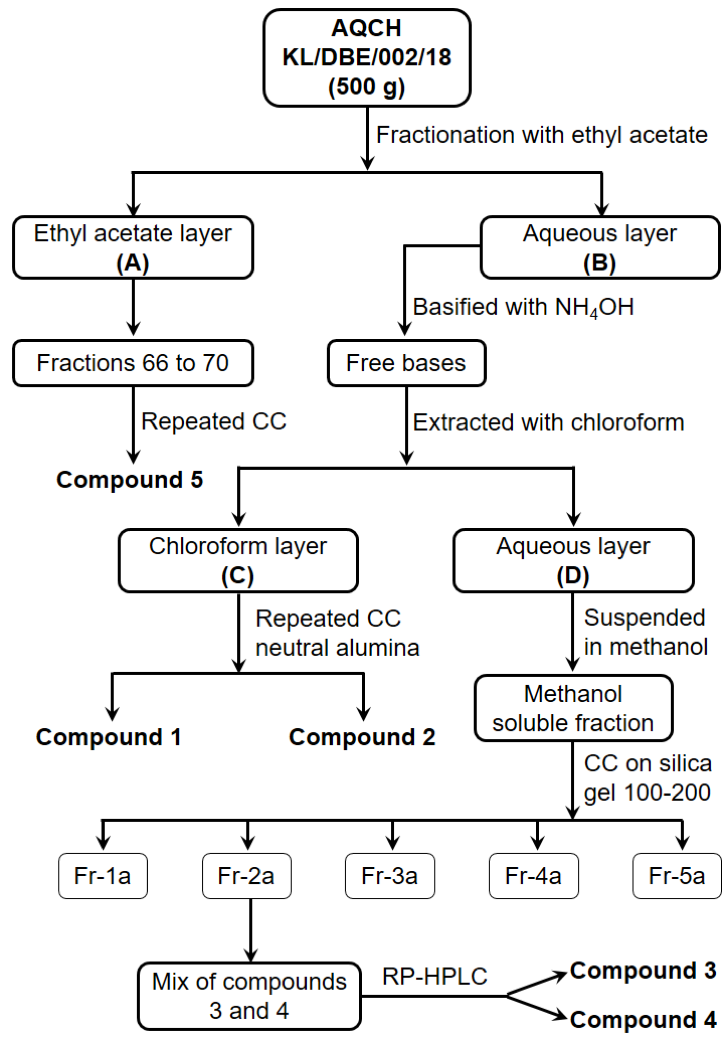


**Supplementary Figure 3: Isolation procedure of marker compounds 1-5 from AQCH**

**Supplementary Table2:** Physico-chemical data of identified markers 1-5 in AQCH

| **Sinococuline (1):** Pink amorphous powder; ^1^H NMR (CD_3_OD, 400 MHz): *δ* 6.74 (1H, d, *J=* 8.4 Hz, H-2), 6.53 (1H, d, *J=* 8.4 Hz, H-1), 4.37 (1H, d, *J*= 5.6 Hz, H-9), 4.28 (1H, d, *J=* 2.8 Hz, H-7), 3.85 (1H, ddd, *J=* 13.3, 3.7 and 3.5 Hz, C-6), 3.82 (3H, s, 3-OCH_3_), 3.69 (3H, s, 8-OCH_3_), 3.15 (1H, dd, *J=* 17.6 and 6.0 Hz, H-10), 2.92 (1H, dd, *J=* 13.3, 3.7 Hz, H-5), 2.88 (1H, d, *J=* 17.7 Hz, H-10), 2.63-2.71 (2H, ddd, *J=* 13.1, 12.5, 3.4 Hz, H-16), 2.16 (1H, t, *J=* 13.2 Hz, H-5), 2.00 (1H, dd, *J=* 12.7, 3.4 Hz, H-15), 1.86 (1H, ddd, *J=* 12.7, 12.5 and 4.7 Hz, H-15); ^13^C NMR (CD_3_OD, 100 MHz): *δ* 147.2 (C-4), 145.9 (C-3), 145.4 (C-8), 131.2 (C-11), 130.2 (C-14), 124.7 (C-12), 119.2 (C-1), 110.5 (C-2), 68.5 (C-6), 66.8 (C-7), 57.4 (3-OCH_3_), 56.7 (8-OCH_3_), 46.7 (C-9), 41.0 (C-16), 39.8 (C-13), 38.4 (C-15), 37.2 (C-5), 36.8 (C-10); ESI-MS *m/z* 334.25 [M+H]^+^ (calcd for C_18_H_24_NO_5_, 334.25). |
| --- |
| **Magnoflorine (2):** Yellowish brown powder; ^1^H NMR (400 MHz, CD_3_OD): *δ*_H_6.72 (1H, d, *J*= 8.0 Hz, H-9), 6.56 (2H, t, *J*= 4.0 Hz, H-3, H-8), 3.84 (3H, s, OCH_3_-10), 3.80 (3H, s, OCH_3_-2), 3.56 (1H, dd, *J*= 18.0, 5.6 Hz, H-6a), 3.45 (1H, dd, *J*= 18.0, 5.2 Hz, H-5), 3.31 (3H, s, N-CH_3_), 3.22 (1H, m, H-4), 3.04 (1H, dd, *J*= 18.0, 5.2 Hz, H-5), 2.89 (3H, s, N-CH_3_), 2.74 (1H, dd, *J*= 20.0, 4.0 Hz, H-4), 2.60 (1H, t, *J*= 13.2 Hz, H-5); ^13^C NMR (100MHz, CD_3_OD): *δ*_C_ 151.9 (C-2), 150.5 (C-10), 149.5 (C-1), 148.6 (C-11), 124.7 (C-7a), 122.4 (C-11b), 122.3 (C-11a), 119.7 (C-6b), 115.6 (C-8), 114.4 (C-3a), 109.5 (C-9), 108.3 (C-3), 70.0 (C-6a), 61.2 (C-5), 55.0 (OCH_3_), 54.7 (OCH_3_), 52.6 (N-CH_3_), 42.2 (N-CH_3_), 30.5 (C-7), 23.4 (C-4); ESIMS *m/z* 343.20 [M+H]^+^ (cal. for C_20_H_24_NO_4_^+^*m/z* 342.20). |
| **20-Hydroxyecdysone (3):** White soft crystals; ^1^H NMR (CD_3_OD, 400 MHz): *δ* 5.81 (1H, d, *J=* 2.6 Hz, H-7), 3.95 (1H, q, H-3α), 3.84 (1H, ddd, *J*= 12.0, 4.0, 3.2 Hz, H-2α), 3.33 (1H, dd, *J=* 11.0, 1.7 Hz, H-22), 3.15 (1H, ddd, *J=* 11.2, 7.0, 2.6 Hz, H-9), 2.39 (1H, dd, *J=* 9.5, 8.0 Hz, H-17), 2.38 (1H, dd, *J=* 13.0, 4.5 Hz, H-5), 2.13 (1H, dt, *J=* 13.0, 13.0, 4.8 Hz, H-12α), 1.99 (1H, H-15α), 1.95 (1H, H-16α), 1.88 (1H, ddd, *J=* 12.8, 4.6, 2.3 Hz, H-12β), 1.81 (1H, H-11β), 1.79 (2H, H-1α/24a), 1.75 (1H, H-4α), 1.73 (1H, H-16β), 1.70 (1H, H-4β), 1.69 (1H, H-11α), 1.66 (1H, H-23a), 1.60 (1H, H-15β), 1.43 (2H, dd, *J=* 13.3, 12.3 Hz, H-1β/24b) 1.28 (1H, dddd, *J=* 13.0, 11.5, 11.0, 4.6 Hz, H-23b) 1.20 (3H, s, 21-CH_3_), 1.20 (3H, s, 26-CH_3_), 1.19 (3H, s, 27-CH_3_), 0.97 (3H, s, 19-CH_3_), 0.89 (3H, s, 18-CH_3_); ^13^C NMR (CD_3_OD, 100 MHz): *δ* 206.4 (C-6), 167.9 (C-8), 122.1 (C-7), 85.2 (C-14), 78.4 (C-22), 77.9 (C-20), 71.3 (C-25), 68.7 (C-2), 68.5 (C-3), 51.8 (C-5), 50.5 (C-17), 48.6 (C-13), 42.4 (C-24), 39.3 (C-10), 37.4 (C-1), 35.1 (C-9), 32.8 (C-4), 32.5 (C-12) 31.8 (C-15), 29.7 (C-26), 28.9 (C-27), 27.4 (C-23), 24.4 (C-19), 21.5 (C-16), 21.5 (C-11), 21.0 (C-21), 18.0 (C-18); HR-MS *m/z* 481.3161 [M+H]^+^ (calcd for C_27_H_45_O_7_, 481.3165). |
| **Makisterone A (4):** White crystals; ^1^H NMR (CD_3_OD, 400 MHz): *δ* 5.81 (1H, d, *J=* 2.6 Hz, H-7), 3.95 (1H, q, H-3α), 3.84 (1H, ddd, *J*= 12.0, 4.0, 3.2 Hz, H-2α), 3.46 (1H, dd, *J=* 11.0, 1.7 Hz, H-22), 3.15 (1H, ddd, *J=* 11.2, 7.0, 2.6 Hz, H-9), 2.39 (1H, dd, *J=* 9.5, 8.0 Hz, H-17), 2.38 (1H, dd, *J=* 13.0, 4.5 Hz, H-5), 2.13 (1H, dt, *J=* 13.0, 13.0, 4.8 Hz, H-12α), 1.99 (1H, H-15α), 1.95 (1H, H-16α), 1.88 (1H, ddd, *J=* 12.8, 4.6, 2.3 Hz, H-12β), 1.81 (1H, H-11β), 1.79 (2H, H-1α/24a), 1.75 (1H, H-4α), 1.73 (1H, H-16β), 1.70 (1H, H-4β), 1.69 (1H, H-11α), 1.60 (1H, H-23a), 1.57 (1H, H-15β), 1.43 (1H, dd, *J=* 13.3, 12.3 Hz, H-1β) 1.29 (1H, dddd, *J=* 13.0, 11.5, 11.0, 4.6 Hz, H-23b), 1.19 (3H, s, 21-CH_3_), 1.17 (3H, s, 27-CH_3_), 1.14 (3H, s, 28-CH_3_), 0.97 (3H, s, 25-CH_3_), 0.94 (3H, s, 19-CH_3_), 0.90 (3H, s, 18-CH_3_); ^13^C NMR (CD_3_OD, 100 MHz): *δ* 204.4 (C-6), 165.9 (C-8), 120.1 (C-7), 83.1 (C-14), 75.9 (C-20), 73.3 (C-22), 71.8 (C-26), 66.6 (C-2), 66.4 (C-3), 49.6 (C-5), 48.2 (C-17), 39.7 (C-10), 37.2 (C-1), 35.3 (C-9), 33.0 (C-12), 32.3 (C-4), 30.9 (C-15), 30.4 (C-13), 29.7 (C-15), 28.7 (C-23), 28.6 (C-24), 25.8 (C-27), 23.8 (C-28), 22.5 (C-19), 19.5 (C-16), 19.3 (C-11), 19.0 (C-21), 16.1 (C-18), 13.2 (C-25); HR-MS *m/z* 495.3318 [M+H]^+^ (calcd for C_28_H_47_O_7_, 495.3322). |
| **Coniferyl alcohol (5):** White solid; ^1^H NMR (CD_3_OD, 400 MHz): *δ* 7.01 (1H, d, *J=* 1.8 Hz, H-3), 6.85 (1H, dd, *J*= 7.9 and 1.8 Hz, H-5), 6.74 (1H, d, *J=* 7.9 Hz, H-6), 6.50 (1H, d, *J*= 15.9 Hz, H-7), 6.20 (1H, dt, *J*= 15.9 and 5.4 Hz, H-8), 4.21 (2H, d, *J*= 6.1 Hz, H-9), 3.87 (3H, s, 2-OCH_3_); ^13^C NMR (CD_3_OD, 100 MHz): *δ* 149.2 (C-2), 147.6 (C-1), 132.2 (C-7), 130.7 (C-4), 127.2 (C-8), 121.1 (C-5), 116.3 (C-6), 110.8 (C-3), 64.0 (C-9), 56.5 (C-2-OCH_3_) |

**Supplementary Table3:** Stability data of AQCH and AQCH tablets with respect to content of Magnoflorine as analytical marker

| **AQCH Extract/ Tablet (Batch ID)** | **Storage condition** | **Magnoflorine content (% w/w)** | | | |
| --- | --- | --- | --- | --- | --- |
|  |  | **1 Month** | **2 Months** | **3 Months** | **6 Months** |
| **Extract**  **(FCH1901002)** | 40±2 ^o^C, 75±5% RH | 0.39 | 0.43 | 0.41 | nd* |
|  | 30±2 ^o^C, 65±5% RH | nd* | nd* | 0.41 | 0.34 |
| **Tablet 100 mg**  **(RYP(6665)079A)** | 40±2 ^o^C, 75±5% RH | 0.37 | 0.37 | 0.38 | 0.43 |
|  | 30±2 ^o^C, 65±5% RH | nd* | nd* | 0.38 | 0.42 |
| **Tablet 300 mg (RYP(6665)079B)** | 40±2 ^o^C, 75±5% RH | 0.41 | 0.4 | 0.41 | 0.45 |
|  | 30±2 ^o^C, 65±5% RH | nd* | nd* | 0.41 | 0.46 |
| **Tablet 500 mg**  **(RYP(6665)079C)** | 40±2 ^o^C, 75±5% RH | 0.4 | 0.4 | 0.41 | 0.45 |
|  | 30±2 ^o^C, 65±5% RH | nd* | nd* | 0.41 | 0.46 |

*nd: not determined; these specific storage conditions were for long-term stability studies and were therefore not sampled on 1^st^ and 2^nd^ month of storage

**Supplementary Protocol 1**

**Supplementary Protocol 1.1 Determination of Magnoflorine content in AQCH drug substance**

Magnoflorine content in AQCH substance was determined using a validated method. Magnoflorine standard procured from sigma Aldrich was used for quantification. A known concentration of Magnoflorine of about 12 ppm was prepared in diluent containing aqueous solution of sodium chloride (1.1% w/w) and water in 80:20 ratio (% v/v). A known concentration of tablet containing about 2400 ppm of aqueous extract was extracted in the same solvent. Both standard and sample were analyzed by high pressure liquid chromatography. 0.1% w/v formic acid in a solution containing water and acetonitrile (Mobile phase A) and a solution containing Mobile phase A and acetonitrile in 30:70 (% v/v) were used in the gradient method. X Select HSS C18 (250 x 4.6)mm , 3.5µm was used. Flow rate was 1.0 mL/min and UV detector of 254nm was used. The method was validated for specificity, precision, accuracy, linearity and stability in analytical solution. Correlation coefficient was found to be 0.99994 when linearity was established from 0.7 ppm to 24.6 ppm of Magnoflorine. % RSD was found to be 0.8% and 0.4% in system precision and method precision study. Recovery study was performed in three replicates at three different level and the % Recovery values were found to be 97.4% to 100.0%. standard solution and sample solution were found to be stable for 60 hours and 54 hours respectively.

**Supplementary Protocol 1.2Quantification of DENV RNA in serum/plasma of AG129 mice by Real Time PCR**

Viral RNA was purified from serum using QiAamp Viral RNA Mini Kit (Qiagen, Cat No. 52904) as per manufacturers protocol. cDNA was prepared from purified RNA using iScriptTM Select cDNA Synthesis Kit (Bio-Rad, Cat No. 1708897) with DENV-2 specific reverse primer as per manufacturers protocol. cDNA was subsequently subjected to qPCR using forward and reverse primers targeting DENV-2 5’ untranslated region (UTR)/Capsid region in conjunction with iTaq Universal SYBR Green Super Mix (BioRad, Cat No. 1725124) in a StepOnePlus Real-Time PCR system (Applied Biosystems). In vitrotranscribed synthetic DENV-2 5’UTR/Capsid RNA was analyzed as standard, in parallel, to calculate DENV-2 viral RNA copies/ml of serumas reported previously (Shukla et al., 2020). Forward and reverse primers used were 5’-AGTTGTTAGTCTACGTGGACCGA-3’ and 5’-CGCGTTTCAGCATATTGAAAG-3’, respectively.

**Supplementary Protocol 1.3Quantification of DENV RNA in small intestinal tissue of AG129 mice by Real Time PCR**

Small intestines were collected from experimental AG129 mice (n=3) and immersed immediately in RNALater RNA stabilization Reagent (Invitrogen, AM7020). Total RNA was purified from intestinal tissue using RNeasy Plus Mini Kit (Qiagen, Cat No. 74134) as per manufacturers protocol. Purified RNA was reverse transcribed and amplified in a single step using SuperScript III Platinum One step Quantitative RT-PCR System with ROX (Invitrogen, Cat No. 11745-100). Forward and reverse primers used for DENV quantification were 5’-CATATTGACGCTGGGAAAGA-3’ and 5’-AGAACCTGTTGATTCAAC-3’, respectivelyas described previously (Shukla et al., 2020).Taqman probe used in DENV quantification was 5’-CTGTCTCCTCAGCATCATTCCA GGCA-3’, tagged with 6-FAM (Dye at 5’) and TAMRA (Quencher at 3’). 18S rRNA served as an endogenous control, which was amplified using 5’- CGGCTACCACATCCAAGGAAG-3’ forward primer and 5’- GCTGGAATTACCGCGGCTG -3’ reverse primer. 6-FAM (Dye at 5’) and TAMRA (Quencher at 3’) tagged Taqman probe used for quantifying 18S rRNA was 5’- TGCTGGCACCAGACTTGCCCTC -3’.

**Supplementary Protocol 1.4Quantification of Vascular Leakage and cytokine levels in small Intestine of AG129 mice**

Vascular leakage was assessed using Evans blue dye which serves as a marker for albumin extravasation. Briefly, 100 μl of 1% Evans blue solution was injected intravenously into the mice, as reported elsewhere(Watanabe et al., 2015; Shukla et al., 2020). Two hr post injection of dye, animals were euthanized and extensively perfused with sterile PBS. For quantitative determination of vascular permeability, tissues were harvested and weighed prior to dye extraction using N,N-dimethylformamide (10 ml/gm of tissue wet weight) at 37 °C for 24 hr and absorbance was read at 620 nm. Vascular leakage data is represented as fold increase in OD_620_ per gm of tissue’s wet weight in comparison to the uninfected control. For cytokines estimation, harvested small intestinal tissue was homogenized in PBS using a Polytron homogenizer. It was centrifuged to collect the clear supernatant for analyzing the levels of TNF-α and IL-6. These cytokines were measured using commercially available ELISA based kits (Invitrogen, Cat No. KMC3011 and KMC0061, respectively).
